# Recruitment Strategies, Response Rates, and Non-Response Patterns in a Nationwide Registry-Based PRO Survey of Cancer Survivors and the General Population: The SURV-ICE Cohort

**DOI:** 10.3390/cancers18101516

**Published:** 2026-05-08

**Authors:** Kristjana Sigurðardóttir, Nanna Friðriksdóttir, Nanna Margrét Kristinsdóttir, Lára Kristjánsdóttir, Hjalti Gunnlaugur Skúlason, Álfheiður Haraldsdóttir, Sigríður Ása Alfonsdóttir, Anna Kristín B. Jóhannesdóttir, Helgi Birgisson, Helga Tryggvadóttir, Freyja Birgisdóttir, Heiðdís Valdimarsdóttir, Thor Aspelund, Lonneke van de Poll-Franse, Sigríður Gunnarsdóttir

**Affiliations:** 1 Icelandic Cancer Registry and Research Center at the Icelandic Cancer Society, 105 Reykjavik, Iceland; 2 Faculty of Nursing and Midwifery, University of Iceland, 102 Reykjavik, Iceland; 3 Department of Oncology, Landspitali-University Hospital, 105 Reykjavik, Iceland; 4 Department of Psychology, Reykjavík University, 102 Reykjavik, Iceland; 5 Centre of Public Health Sciences, University of Iceland, 102 Reykjavik, Iceland; 6 Division of Psychosocial Research and Epidemiology, Netherlands Cancer Institute, 1066 CX Amsterdam, The Netherlands; 7 Department of Medical and Clinical Psychology, Tilburg University, 5000 LE Tilburg, The Netherlands

**Keywords:** cancer survivorship, patient-reported outcomes, health-related quality of life, registry-based study, survey response rates, mixed-mode survey, health literacy, population-based

## Abstract

**Background**: Population-based studies using patient-reported outcomes provide important insights into health-related quality of life (HRQoL) and the long-term impact of cancer and its treatment. Declining response rates and potential non-response bias have presented methodological challenges, particularly in registry-based surveys including both cancer survivors and population controls. The SURV-ICE study was established to assess HRQoL and health literacy among cancer survivors in Iceland while evaluating recruitment processes and participation patterns in a nationwide mixed-mode survey. **Methods**: SURV-ICE is a nationwide, population-based cross-sectional survey conducted in Iceland in 2025. Adults diagnosed with invasive cancer between 2014 and 2024 were identified through the Icelandic Cancer Registry and invited alongside an age- and gender-matched control group sampled from national registers. Participants completed validated EORTC instruments. Recruitment used a mixed-mode strategy including postal invitations, web-based and paper questionnaires, electronic reminders via the national health portal, and targeted telephone follow-up. **Results**: Among 10,005 cancer survivors 5489 (54.9%) responded to the questionnaires and among 5663 controls 2297 (40.6%) responded to the questionnaires. In both groups participation peaked among individuals aged 60–79 years and was lowest among the youngest and oldest groups. Cancer survivors had higher odds of participation than controls (OR 1.84; 95% CI 1.72–1.97). Most responses followed the initial postal invitation and first electronic reminder. Data completeness was high (≥80% item completion in 95.7% of instruments). **Conclusions**: Nationwide registry-based recruitment for large-scale PRO research is feasible in Iceland and can achieve competitive response rates without incentives. SURV-ICE provides an infrastructure for future research on cancer survivorship, HRQoL, and health literacy.

## Simple Summary

Understanding how cancer and its treatment affect people’s daily lives is an important part of improving long-term care for cancer survivors. Studies that collect information directly from patients can provide valuable insight into quality of life, symptoms, and other health concerns after cancer treatment. However, obtaining good participation in large national surveys can be challenging. The SURV-ICE study invited cancer survivors across Iceland, along with individuals from the general population to complete questionnaires about health-related quality of life, symptoms and health literacy. The study achieved strong participation using a combination of postal invitations, online questionnaires, and follow-up reminders. Participation was higher among cancer survivors than among the general population, and most responses were received early in the recruitment process. The results show that large national surveys collecting patient-reported information are feasible in Iceland and can provide an important resource for future research on cancer survivorship and long-term health outcomes.

## 1. Introduction

Population-based studies using patient reported outcomes (PROs) provide essential information on health-related quality of life (HRQoL) and the broader impact of cancer and its treatment on patients’ lives [1,2]. As cancer survival continues to improve [3], representative PRO data from both cancer survivors and the general population are increasingly needed to support epidemiological research, benchmark survivorship outcomes, and inform health services planning [1]. In clinical and research settings, PRO measures capture symptoms, functioning, and well-being that may not be reflected in clinical records, and they can support evaluation of care quality and the long-term consequences of cancer and treatment [4,5,6]. Despite this growing emphasis, population-based PRO data across cancer types remain limited in Iceland, and participation patterns in nationwide registry-based PRO surveys in this context are not well described.

Population-based PRO studies face important methodological challenges, most notably declining response rates and the rise in non-response bias [7,8,9]. Increased reliance on online data collection methods may introduce additional barriers, as participation depends on digital access and literacy [10]. Low participation rates may reduce statistical power and introduce systematic selection bias, thereby limiting the validity and generalizability of findings [8]. These concerns are particularly relevant in studies including both cancer and non-cancer populations where differential response by disease status, age, or socioeconomic factors may compromise comparability between groups [11]. Furthermore, response rates in registry-based and mixed-mode surveys vary considerably across countries, cohort characteristics, and data collection methods. Survey quality is increasingly judged not solely by response rate but by whether potential non-response bias can be evaluated and addressed [7,8,11,12,13,14].

To mitigate these challenges, mixed-mode data collection strategies and structured follow-up procedures are widely recommended [15,16,17,18]. Offering multiple response modes may improve accessibility and participation across population subgroups [11,15,19,20,21] and recruitment strategies tailored to participant characteristics may further enhance response (e.g., telephone follow-up among older adults) [10]. In addition, use of trusted institutional platforms may increase perceived legitimacy and engagement, particularly in large-scale studies where direct clinician recruitment is not feasible [10,15,22,23]. However, empirical evidence on recruitment efficiency and response patterns in nationwide registry-based cancer survivorship PRO surveys remain scarce.

The SURV-ICE study was established as a nationwide, population-based cross-sectional survey to assess HRQoL and health literacy among cancer survivors, alongside a general population comparison group. Beyond its substantive aims, SURV-ICE provides a unique opportunity to evaluate recruitment processes, response accumulation, and participation patterns in a registry-based mixed-mode PRO survey at national scale.

Iceland represents a particularly suitable setting for this work. Unique personal identification numbers enable accurate identification of eligible individuals and linkage across datasets, and nationwide cancer registration through the Icelandic Cancer Registry (ICR), established in 1954 and mandated by law, is estimated to capture 99.9% of all cancer diagnoses [24]. In addition, linkage to demographic registers (Statistics Iceland) and clinical registers (Directorate of Health) strengthens the SURV-ICE dataset and enables responders/non-responder comparisons using registry variables. To date, no nationwide study in Iceland has systematically collected PROs across cancer types and stages with linkage to high-quality national registers.

The primary aim of this article is to describe the recruitment strategy and response rates of the SURV-ICE cohort and examine differences in key sociodemographic and registry characteristics between responders and non-responders in both cancer and general population samples. A secondary aim is to introduce SURV-ICE as a research resource for future studies of cancer survivorship, HRQoL, and related outcomes.

## 2. Materials and Methods

### 2.1. Study Design, Population and Sampling Frame

SURV-ICE is a nationwide, population-based cross-sectional study of adults residing in Iceland. The study includes three groups: (1) cancer survivors who self-reported being more than 12 months beyond completion of their most recent treatment, including breast cancer survivors who were >12 months post-treatment but still receiving maintenance treatment; (2) cancer survivors who self-reported having received cancer treatment within the past 12 months, as well as prostate cancer survivors who had not received treatment but were under active monitoring; and (3) a population-based comparison group with no history of cancer prior to the study period.

**Figure 1:**
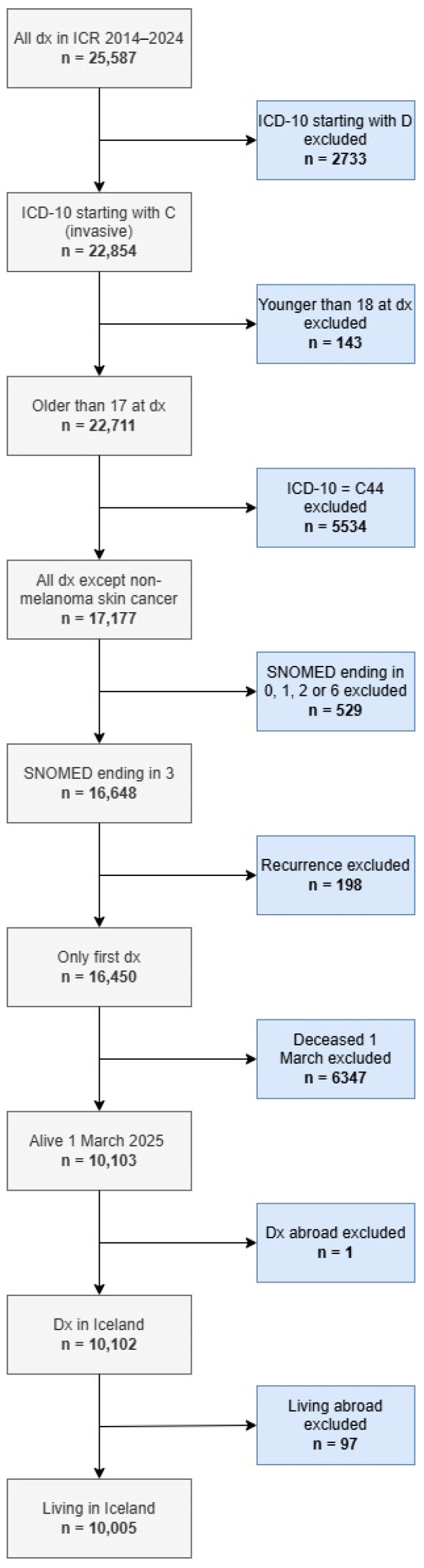
Flowchart showing exclusion process for identification of eligible cancer cases from the Icelandic Cancer Registry (2014–2024).

Eligible participants in the cancer cohort were adults ≥18 years at diagnosis, with no upper age cutoff, diagnosed with invasive cancer between 1 January 2014 and 31 December 2024. As data collection occurred in 2025, no invited individuals were aged 18 years at the time of survey. Information on cancer diagnoses was obtained from the ICR. Treatment phase (≤12 months vs. >12 months since most recent treatment) was determined by self-report at survey entry and used for questionnaire allocation. Individuals with non-invasive cancers, non-melanoma skin cancers, cancers representing recurrence of a primary malignancy diagnosed prior to 1 January 2014, or those deceased or not residing in Iceland as of 1 March 2025, were excluded. Following application of the exclusion criteria, the final sampling frame for the cancer cohort comprised 10,005 individuals (Figure 1).

The comparison group was randomly sampled from Register Iceland and frequency-matched to the cancer cohort by age and gender. Of these, 337 were excluded due to a cancer diagnosis prior to 2014, resulting in a final control sample of 5663 individuals. All eligible individuals meeting inclusion criteria during the study period were invited. Power calculations conducted for the overall SURV-ICE research program indicated that the anticipated sample size would provide adequate statistical power for planned analyses of the cohort. The sampling frame was constructed in February 2025. The study is reported in accordance with the STROBE guidelines for cross-sectional studies [25].

### 2.2. Questionnaires and Measures

HRQoL was measured using validated instruments developed by the European Organization for Research and Treatment of Cancer (EORTC) [26,27]. Questionnaire allocation was determined based on cancer diagnosis obtained from the ICR and self-reported current treatment status. Participants who had received cancer treatment within the past 12 months were administered the EORTC QLQ-C30 [28], supplemented with relevant disease-specific modules for breast (QLQ-BR42) [29], prostate (QLQ-PR25) [30], or colorectal cancer (QLQ-CR29) [31,32,33].

Participants who had completed treatment more than 12 months prior to the survey were administered the EORTC Quality of Life Cancer Survivorship Questionnaire (QLQ-SURV100) [34], together with corresponding site-specific survivorship modules (QLQ-BR-SURV40, QLQ-PR-SURV19, and QLQ-CR-SURV33). The QLQ-SURV100 recently completed international field testing (Phase IV) and cross-cultural validations [34,35]. Versions in Icelandic, English and Polish were used in accordance with EORTC translation procedures [26].

Participants in the general population comparison group were administered the EORTC QLQ-C30 and a modified version of the QLQ-SURV100, excluding 30 cancer-specific items. Excluding cancer-specific items ensured face validity in the general population while preserving comparability on shared domains such as physical, role, emotional, cognitive, and social functioning, as well as fatigue, pain and global health status.

Health literacy was measured for all participants with the validated 16-item European Health Literacy Survey Questionnaire (HLS-EU-Q16) [36].

In addition, survivors who were more than 12 months post-treatment were administered a 17-item binary, study-specific needs assessment. This checklist was developed for the study and aligned with key survivorship domains of the QLQ-SURV100 to evaluate patients perceived need for professional support across key post-treatment domains. For each need, participants indicated whether they received sufficient assistance, enabling classification as met or unmet. The items cover a wide range of common concerns, including physical, emotional, cognitive, social, sexual, practical, financial, and family-related issues, as well as fears of recurrence, fertility considerations, changes in health behaviors, and returning to work.

**Figure 2:**
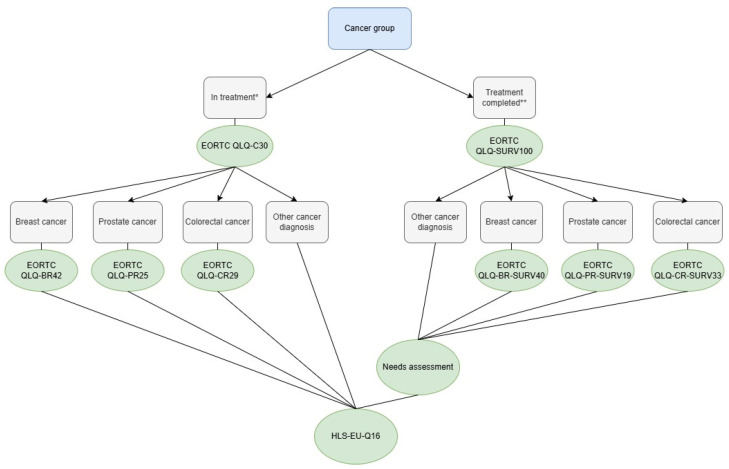
Flowchart illustrates the allocation of questionnaires to cancer survivors. * Note. Cancer survivors that received treatment within the past 12 months and prostate cancer survivors who had not received treatment but were under active monitoring. ** Note. Cancer survivors > 12 months beyond completion of treatment and breast cancer survivors > 12 months post-treatment receiving maintenance treatment.

The total number of questionnaire items varied according to study group and cancer type, ranging from 46 to 160 items, with an estimated completion time of 20–40 min. To enhance accessibility and inclusivity, questionnaires were available in Icelandic, English, or Polish, in line with best practices in population-based survey research [37]. Those languages were chosen as the largest immigration group in Iceland is Polish [38]. An overview of questionnaire allocation is presented in Figure 2.

### 2.3. Recruitment Procedures and Contact Strategy

Data collection began in March 2025. All sampled individuals, both cancer survivors and controls, were sent a postal invitation letter to their registered home address as recorded in Registers Iceland. The letter included study information and a personal login password enabling access to the web-based questionnaires administered by the research company Gallup [39] via a secure survey platform. Participants could request a paper version by telephone or email, consistent with evidence supporting mixed-mode data collection to improve response rates [18,19,40,41,42].

Follow-up for the postal invitation was conducted primarily through Heilsuvera [43], Iceland’s official secure national health portal operated by the Directorate of Health. Individuals registered in Heilsuvera received a text message to personal phone numbers, containing a direct link to the study materials, enabling access to study information, electronic consent, and questionnaire completion without additional log-in requirements. The same message was also accessible through the Heilsuvera portal interface. According to the Directorate of Health (personal communication, March 2026), 89.3% of individuals 18 years and older living in Iceland accessed this portal in 2025 (the year of data collection).

Telephone follow up was prioritized for older non-responders (primarily aged 60–80 years) as part of an age-stratified follow-up strategy designed to optimize response rates. This approach is consistent with evidence suggesting that direct contact methods may be more effective than electronic reminders among older adults [10,11].

#### Data Collection Timeline

The National Bioethics Committee approved up to three contact attempts per participant. Initial follow-up consisted of a text message via Heilsuvera to non-responders. Subsequent follow-up strategies were adapted according to age group: participants aged 18–60 years received additional text reminders, while those aged 60–80 were contacted by telephone. During the final follow up, remaining eligible non-responders who had not yet reached the maximum number of contact attempts were contacted by either a text message (≤80 years) or a phone call (81–85 years). Participants aged ≥ 86 years received a reduced number of contact attempts compared with the approved maximum. This deviation from the approved maximum was implemented to reduce burden following feedback from relatives and was applied consistently by age group.

Data collection closed on 6 November 2025. Most responses were received by the end of August; however, the survey platform remained open until November to allow return and processing of paper-based questionnaires. Response accumulation following each contact stage was examined descriptively.

### 2.4. Public Information and Media Outreach

In parallel with the invitation process, general information about the study was made publicly available through institutional channels and national media. Researchers appeared in interviews on television, on radio shows, and in online and print media introducing the study. These activities were not directed at individual participants and were not considered part of the formal contact strategy. Targeted messages were shared by the Icelandic Cancer Society on their website and social media platforms and included, among other material, messages from nationally well-known cancer survivors. Similar messages were shared by Kraftur, a national patient support organization for young adult cancer survivors, utilizing tailored messages from their members on social media platforms. In addition, the study was advertised with banners on the most used online media platforms in Iceland. Interviews and messages on social media and online advertising were lined up with contact with participants, with most of the media coverage at the initiation of the study, and social media and online advertisements lined up with follow-up contacts. In addition to PR strategies aimed at the public, advertising aimed at cancer patients receiving treatment were made visible in clinical settings. Again, not targeting individuals.

### 2.5. Data Management

Web-based questionnaires were administered by the research company Gallup using Forsta’s online survey platform. Gallup managed questionnaire distribution and data collection and provided raw datasets to the research team. Paper questionnaires were distributed by the research team, and returned completed questionnaires were manually entered by trained staff at the Icelandic Cancer Society. No questionnaire responses were stored within Heilsuvera. All data were collected and securely stored within Gallup’s secure research system and, after data collection was completed, delivered via secure methods to the research team for storage at the Icelandic Cancer Society.

The SURV-ICE cohort was constructed by linking questionnaire data to national register data using unique personal identification numbers. Data management and documentation adhered to the FAIR (Findability, Accessible, Interoperable, Reusable) principles [44], including structured metadata and variable descriptions to support transparency, reproducibility, and responsible future reuse of the data.

Due to legal and ethical constraints, including the General Data Protection Regulation and national data protection legislation, individual-level data cannot be publicly shared. However, aggregated, non-identifiable data may be made available for research purposes under controlled conditions. Access to individual-level research data may be granted to researchers who obtain the required ethical and institutional approvals and collaborate with members of the SURV-ICE research team.

### 2.6. Statistical Analysis

Data management and statistical analyses were performed using Python (version 3.13.7; Python Software Foundation, Wilmington, DE, USA) and R (version 4.5.2, R Foundation for Statistical Computing, Vienna, Austria), implemented in RStudio (version 2026.01). Data completeness and quality were assessed by examining completion of survey instruments (questionnaires) and item-level responses across all survey questions. This is a response-rate methodological analysis with statistical analyses focusing on describing response rate, response accumulation over time, and differences between responders and non-responders. Response rate was calculated as the proportion of completed questionnaires among all eligible invited individuals.

The primary outcome was response status (responder vs. non-responder). To assess potential non-response bias, basic demographic and registry variables available for the entire sampled population were compared between responders and non-responders. Comparisons based on gender, age group, region of residence, and study group were initially evaluated using chi-square tests. These variables were available for the entire sampled population from registry sources at time of sampling and therefore constituted the only variables suitable for responder vs. non-responder comparisons. Associations between these variables and response status were further examined using logistic regression models, with results presented as odds ratios (ORs) with 95% confidence intervals (CIs).

### 2.7. Ethical Consideration

The study was approved by the National Bioethics Committee (VSN-2410028) on 23 December 2024.

Informed consent was obtained electronically or in a paper form. The consent included permission to use questionnaire data and to link responses with relevant national registers. Optional consent was sought for future follow-up contact and genetic data linkage. Additional approvals for registry linkage were obtained from the Directorate of Health and the National University Hospital of Iceland.

## 3. Results

### 3.1. Study Population and Sampling Outcomes

A total of 15,668 individuals were invited to participate in the SURV-ICE study, including 10,005 cancer survivors and 5663 population controls.

Baseline demographic and registry characteristics of invited individuals are shown in Table 1. The cancer cohort and the population controls were comparable with respect to age, gender, and region of residence.

Within the other cancer category, the largest diagnostic groups were lung (5.1%), kidney (4.4%), malignant melanoma (4.2%), rectal (3.1%), corpus uteri (2.8%), non-follicular lymphoma (2.1%), and thyroid cancer (2.0%). All other cancer types each represented less than 2% of the cancer cohort.

### 3.2. Response Rates by Age Study Group (Cancer vs. Control)

Response rates stratified by study group and age category are presented in Table 2.

Participation differed significantly between the study groups. Response among individuals diagnosed with cancer was 54.9%, compared with 40.6% among population controls (χ^2^ test, *p* < 0.001). Among responding cancer survivors 65.3% reported having completed treatment more than 12 months prior to participation, while 34.7% reported treatment within the previous 12 months.

Among participants aged 19–80 years, response rates were 59.2% in the cancer group and 42.7% in the control group. In contrast, response rates were substantially lower among individuals aged 81–99 years across both groups (30.5% in the cancer group and 27.5% in controls). Within the cancer cohort aged 19–80 years, participation varied by diagnosis, with the highest response observed among breast cancer survivors (67.4%), followed by prostate (58.9%), colorectal (58.8%) and other cancers (55.6%).

### 3.3. Response Accumulation by Contact Stage

**Figure 3:**
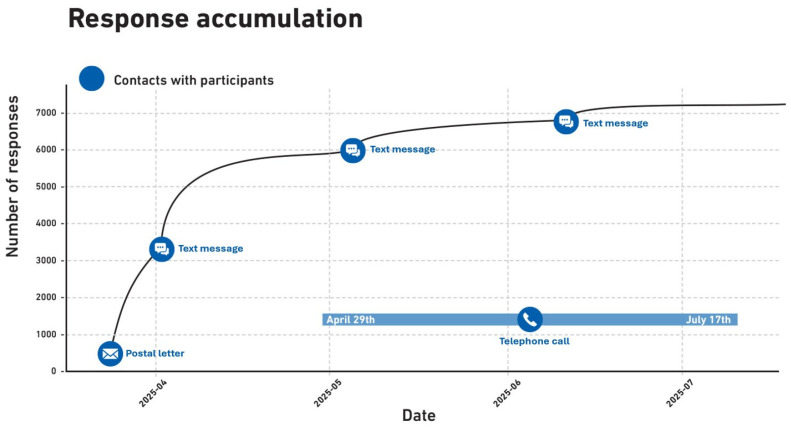
Incremental contribution of successive recruitment contacts to cumulative response.

Participation accumulated progressively across recruitment stages (Table 3). The initial postal invitation resulted in 3437 completed questionnaires (44.1% of total response). The first follow-up text messages via Heilsuvera increased cumulative responses to 6268 (80.5% of total responses). Subsequent age-stratified follow-up, including text messages and telephone calls, yielded progressively smaller increases in participation, ultimately reaching a total of 7786 completed responses (Figure 3). Telephone contact was limited to individuals with available phone numbers, and not all attempts resulted in direct contact due to unanswered calls or unavailable numbers.

The study team received approximately 500 telephone inquiries regarding the study, most following the initial postal invitation and primarily requesting paper-based questionnaires. In response, 458 paper questionnaire packets were distributed, of which 237 (52%) were returned and completed. Paper respondents were predominantly aged 70 years and older.

### 3.4. Mode of Completion

Of the 7786 completed questionnaires, 7535 (96.8%) were submitted via the web-based platform. Paper questionnaires accounted for 237 responses (3.0%), 13 participants completed the survey by telephone and one participant responded in person. Mode of completion differed by age, with paper questionnaires used primarily by older participants: 89.6% of paper responses were from individuals aged 70 years and older.

### 3.5. Comparison of Responders and Non-Responders

Comparisons between responders and non-responders within each study group are presented in Table 4.

In the cancer cohort, 5489 individuals (54.9%) participated, compared with 2297 individuals (40.6%) in the population control group. Within the cancer cohort, participation differed significantly by gender, age group, and cancer type (all *p* < 0.001), whereas regional differences did not reach statistical significance (*p* = 0.051). In the control group, participation varied significantly by age (*p* < 0.001) and region (*p* = 0.014), but not by gender (*p* = 0.824).

Across both groups, the lowest participation was observed among individuals aged 19–39 years and 80–99 years and highest among those aged 60–79 years.

### 3.6. Multivariable Analysis of Participation (Cancer vs. Control)

In multivariable logistic regression analyses adjusting for gender, age group, and region, individuals diagnosed with cancer had statistically significant higher odds of participation compared with population controls (OR = 1.84; 95% CI 1.72–1.97), corresponding to an 84% increase in the odds of participation.

Stratified multivariable analyses conducted separately among individuals diagnosed with cancer and population controls (Table 5) showed that, males with cancer had 14% lower odds of participation compared with females, whereas no statistically significant gender difference was observed among population controls.

Participation was associated with age in both groups. Among individuals diagnosed with cancer, compared with the 70–79-year age group, lower odds of participation were observed in the youngest (19–39 years) and oldest (80–99 years) age groups (19% and 64% lower odds, respectively), while individuals aged 60–69 years had 24% higher odds of participation. Among population controls, compared with the 70–79-year age group, individuals in the youngest two (19–39 years; 40–49 years) age groups had 61% and 26% lower odds of participation, respectively. Furthermore, among the oldest age group (80–99 years), 48% lower odds of participation were observed while those aged 60–69 years had 15% higher odds of participation, compared with the 70–79-year age group.

Compared with the group of other cancers, individuals with prostate and breast cancer had 25% and 45% higher odds of participation, respectively, whereas colorectal cancer diagnosis was not significantly associated with participation.

### 3.7. Data Quality and Completeness

Overall, 98.0% of participants completed ≥ 80% of items in at least one assigned instrument (Table 6), while 95.7% of all administered instruments had ≥80% of items completed (Table 7).

## 4. Discussion

The SURV-ICE study established a nationwide, population-based cohort integrating PROs with high-quality registry data. The study demonstrated the feasibility of large-scale mixed-mode recruitment within a registry-based cancer survivorship survey and achieved a strong overall response rate.

In comparable Nordic registry-based PRO studies on cancer patients, response rates have ranged from 34 to 70% [45,46,47,48,49]. Higher response rates in this range largely reflect earlier studies, whereas more recent population-based surveys have experienced substantial declines in participation [7,47]. Response rates among control populations in similar studies have ranged from 19 to 35% [45,46,47,49]. In this context, the SURV-ICE response rate of 54.9% among cancer survivors falls within the upper range of contemporary studies. The 40.6% response among controls also compares favorably with recent population-based surveys. Previous studies have typically relied on single-mode data collection (either postal or electronic) and more uniform follow-up strategies, which may limit accessibility across demographic groups [10,11,15,19]. In contrast, SURV-ICE implemented a mixed-mode recruitment approach combined with age-tailored follow-up procedures and integration with a national electronic health platform. These features likely contributed to improved participation, particularly among older individuals who may be less responsive to digital-only approaches. However, such strategies also introduce potential trade-offs, including increased logistical complexity and the possibility of mode effects between response formats. Overall, these findings indicate that registry-based recruitment combined with tailored follow-up strategies as well as mass media campaigns can achieve competitive participation levels without financial incentives.

Participation differed significantly between study groups, with individuals diagnosed with cancer demonstrating higher engagement than controls. This pattern likely reflects topic salience, as personal relevance is a well-established determinant of survey participation [8,15]. Given the clinical and societal impact of cancer, higher engagement among cancer survivors is consistent with prior survey research [7,20,50,51,52], and has also been observed in large EORTC QLQ-C30 normative data collections, including national reference data from the Netherlands and international datasets [53,54]. Although participation in the general population sample was lower, it remained well within acceptable ranges for population-based surveys conducted without incentives. Informal feedback during data collection indicated that many responders perceived the study topic as important and appreciated the opportunity to contribute.

The distribution of cancer types in the study reflected both cancer incidence and survival in Iceland. Breast and prostate cancers, the most common types of cancers in Iceland [55], were among the most represented diagnoses as expected, given their high incidence and survival rates. These groups also had higher adjusted odds of responding than survivors in the other cancer group. In contrast, survivors of cancers with poorer prognosis, including lung, pancreas, liver and brain cancers, may be less represented, since fewer individuals survive long enough or are well enough to participate although the response rate ranged at 50% for those cancers. As a result, cohort estimates may therefore more strongly reflect the outcomes of common cancer types with higher survival. Future analyses should account for cancer type and conduct cancer-specific analyses where sample size allows.

Response rates exhibited a clear age-related gradient. Participation was highest among individuals aged 60–79-years and declined in both younger (19–39 years) and older (80–99 years) groups, especially among population controls. Despite high digital access and literacy, lower participation among younger individuals may reflect lower perceived relevance, competing time demands, or reduced engagement with survey-based research. While participation among the oldest age group was lower, the observed rate remained comparatively strong for a population traditionally underrepresented in survey research [56]. Women were modestly more likely to participate than men within the cancer cohort, consistent with prior survey research [7,8], while no significant gender differences were observed within the general population group.

Recruitment accumulation followed a characteristic cumulative response pattern, with most responses obtained after the initial postal invite. Subsequent contacts yielded progressively smaller incremental gains, aligning with established evidence of response rates typically plateauing after the primary contact waves [50,57].

Several design factors likely contributed to the observed participation levels. First, population-based research is generally well received in Iceland [58,59]. Second, the mixed-mode strategy, combining an introductory postal invitation letter offering both web-based and paper surveys followed by age-stratified reminders via text messages and telephone calls, appears to have enhanced accessibility across population subgroups, consistent with previous research [11,15,17,18]. Although most responses followed the postal invitation, additional contact modes likely facilitated participation among individuals less likely to respond electronically. Given that older age distribution of the cancer population, offering paper-based response options and limited telephone follow-up may have been particularly important, as digital-only approaches risk excluding older or less digitally literate individuals [10].

Third, the use of Heilsuvera, Iceland’s secure national patient health portal, provided a trusted healthcare context for outreach and may have enhanced perceived legitimacy of the study [22]. Iceland’s relatively high level of digital health integration, further accelerated during the COVID-19 pandemic, may also have supported web-based participation [60,61,62].

Fourth, respondent burden was actively limited by restricting questionnaire length. Completion rates were excellent even among breast cancer survivors who received the highest number of items, indicating necessary sustained engagement throughout survey completion, perhaps related to salience [8,15].

Fifth, the relatively extended timeframe between contact waves allowed participation to accumulate gradually, a more compressed recruitment schedule may have resulted in lower overall response. Initially the plan was to complete data collection in a shorter time but early in the data collection phase it became clear that complete answers continued to cumulate day by day without new follow up. Therefore, the decision was made to extend the data collection timeframe, allowing each new follow-up to reach its full effect.

Cost effectiveness is an important consideration when executing a large-scale study. Although postal letters involve substantial upfront expense, they generated the largest proportion of responses and appear justified given the response levels obtained. Text message reminders were relatively inexpensive and highly efficient, facilitated by widespread use of Heilsuvera, which provided reliable access to registered phone numbers beyond standard directories. In contrast, telephone follow-up was resource intensive and yielded comparatively modest additional responses. Future large-scale surveys may therefore consider even more targeted telephone strategies rather than universal implementation.

Completion rates were high at both the participant and instrument levels, with minimal missing data, supporting the internal validity of the dataset and its suitability for future analyses. Beyond response metrics, the SURV-ICE cohort represents a substantial national research infrastructure. Its nationwide coverage, absence of an upper age limit, and linkage of PRO data with comprehensive registry information, including demographic and clinical variables, create a strong platform for population-based survivorship research, including analyses of health-related quality of life and health literacy.

### 4.1. Strengths and Limitations

The primary strength of the SURV-ICE cohort lies in its nationwide sampling frame, supported by near-complete cancer ascertainment from high-quality national registries. This approach ensured broad inclusion across cancer types and stages. The availability of personal identification numbers also allows cross linkage of PRO data with registry information. High data completeness further supports the overall quality and reliability of the dataset. Also, the response rate among cancer survivors was high (54.9%), with particularly strong participation among breast cancer survivors (62.7%).

An additional strength is the inclusion of an age and gender matched population control group, which enhances interpretability by allowing contextualization of survivorship outcomes against general population norms.

This study has several limitations. As a cross-sectional survey, findings reflect survivorship at a single timepoint, and no causal inferences can be made. Treatment phase was self-reported, which may result in response error.

Although multiple contact strategies were employed, the reduced number of follow-ups among the oldest participants may limit representativeness in this age group. Similarly, the lower response rate in the youngest age group limits the generalizability of the finding to this age cohort. Participation was higher among individuals with cancer than controls, which may introduce differential selection bias and slight overrepresentation of more engaged or health-conscious survivors in comparative analysis. This may affect estimated differences in outcomes between cancer survivors and population controls, particularly if non-responders differ systematically from responders by age or gender. However, most observed differences were modest, suggesting that any impact is likely limited, especially if future analyses adjust for these key predictors.

Lower response rates in specific geographic regions may have been influenced by population displacement as a result of volcanic activity in the study period. Mixed-mode data collection improves accessibility but may introduce modest mode effects between web-based and paper respondents; however, the proportion of paper and telephone responses was small, suggesting that any such effects are likely limited.

The SURV-ICE study demonstrates that large-scale, registry-based PRO research is feasible within a small, highly digitalized, national healthcare system. The combination of comprehensive population registries and widespread access to a national electronic health portal likely contributed to the observed recruitment success and may not be directly replicable in larger or more heterogeneous healthcare systems. Nevertheless, several elements of the study design, including mixed-mode data collection, age-tailored follow-up strategies, and the use of trusted national institutions to facilitate contact and participation, suggest that similar approaches could be implemented in more diverse settings with differing structural and cultural contexts.

### 4.2. Recommendations for Future Research

Future research should aim to build upon the SURV-ICE cohort, and establish follow-up protocols, to assess trajectories of HRQoL and survivorship outcomes over time. Given the international validation of EORTC instruments, cross-country comparisons may provide valuable benchmarking of survivorship outcomes.

Further methodological work should evaluate optimized recruitment strategies for specific demographic subgroups and formally assess potential mode effects between electronic and paper-based responses. The research team made numerous efforts to reach the older age groups including offering the option of paper-based questionnaires and using phone calls rather than text messages for follow-up. More targeted efforts should also have been applied to increase participation in the youngest age group; for example, by using more social media platforms and targeted advertisement.

Given Iceland’s high level of digital health integration, future research may also explore embedding longitudinal PRO monitoring within Heilsuvera or similar high-uptake platforms to support survivorship surveillance. Evidence from randomized trials suggests that response to electronic questionnaires can be substantially improved through brief and visually enticing invitations, monetary or non-monetary incentives, shorter questionnaires, personalization, and emphasizing the societal benefits of participation. However, overall response rates to electronic surveys are traditionally lower than those achieved with postal questionnaires, and offering participants a choice between postal and electronic response has been shown to significantly increase participation [15]. Future PRO studies could evaluate these strategies using mixed-mode methods to optimize recruitment while maintaining representativeness and cost-effectiveness in a survivor population.

Future research should evaluate the transferability of the recruitment strategies identified in this research across different healthcare and cultural contexts. Mixed-mode data collection, reduction in respondent burden, and age-tailored follow-up strategies appear particularly promising and may be adaptable to other settings. The use of trusted national institutions or health platforms may further enhance participation where such infrastructure exists.

## 5. Conclusions

The SURV-ICE study demonstrates that nationwide, registry-based recruitment of cancer survivors and population controls for large-scale PRO research is feasible in Iceland and can achieve competitive response rates without specific incentives. The observed feasibility is likely influenced by Iceland’s integrated national registry infrastructure and high level of digital health uptake and may not be directly generalizable to larger or more heterogeneous healthcare systems. Participation patterns were consistent with established survey methodology literature, including higher engagement among cancer survivors, age-related gradient in response, and diminishing returns across successive contact waves. The mixed-mode recruitment strategy, chosen to reduce barriers to response in line with recommendations to adapt recruitment strategies to the target population and leverage trusted institutional platforms to enhance engagement, proved effective in facilitating participation across population groups. High data completeness and successful linkage to comprehensive national registry data support the internal validity and analytical value of the cohort.

As cancer survivorship continues to increase globally, scalable and methodologically rigorous approaches such as SURV-ICE are essential to ensure that population-level survivorship outcomes are accurately measured and meaningfully interpreted, allowing us to inform future research, policy, and survivorship care planning.

## Figures and Tables

**Table 1:** Baseline characteristics of individuals invited to participate in SURV-ICE study, by study group.

	Cancer Survivors	Population Controls
	(*N* = 10,005)	(*N* = 5663)
**Gender**		
Male	4820 (48.2%)	2740 (48.4%)
Female	5184 (51.8%)	2923 (51.6%)
Non-binary	1 (0.0%)	0 (0%)
**Age**		
19–39	410 (4.1%)	256 (4.5%)
40–49	658 (6.6%)	416 (7.3%)
50–59	1312 (13.1%)	812 (14.3%)
60–69	2619 (26.2%)	1538 (27.2%)
70–79	3232 (32.3%)	1738 (30.7%)
80–99	1774 (17.7%)	903 (15.9%)
**Region of residence**		
Capital region	6371 (63.7%)	3421 (60.4%)
Western region	509 (5.1%)	332 (5.9%)
Westfjords	156 (1.6%)	102 (1.8%)
Northern region	1112 (11.1%)	662 (11.7%)
Eastern region	249 (2.5%)	175 (3.1%)
Southern region	970 (9.7%)	555 (9.8%)
Southern peninsula	629 (6.3%)	399 (7.0%)
Moved abroad *	9 (0.1%)	17 (0.3%)
**Cancer diagnosis**		
Breast	2226 (22.2%)	
Prostate	1934 (19.3%)	
Colorectal	1127 (11.3%)	
Other cancers	4718 (47.2%)	

Note: Data derived from the Icelandic Cancer Registry and Register Iceland, * Note. Some participants were living in Iceland at the time of sampling but moved abroad during the study period.

**Table 2:** Response rates by age and cancer diagnosis.

Study Group	19–99 Years		19–80 Years		81–99 Years	
	*N*	Responses *n* (%)	*N*	Responses *n* (%)	*N*	Responses *n* (%)
**Cancer survivors (all)**	10,005	5489 (54.9)	8488	5027 (59.2)	1517	462 (30.5)
Breast	2226	1395 (62.7)	1933	1302 (67.4)	293	93 (31.7)
Colorectal	1127	589 (52.3)	868	510 (58.8)	259	79 (30.5)
Prostate	1934	1061 (54.9)	1621	955 (58.9)	313	106 (33.9)
Other cancer	4718	2444 (51.8)	4066	2260 (55.6)	652	184 (28.2)
**Control**	5663	2297 (40.6)	4896	2087 (42.7)	767	210 (27.5)

Note: *N* represents the number of invited individuals in each category.

**Table 3:** Recruitment stages, contact modes, and response accumulation in the SURV-ICE study.

Stage	Contact Mode	Target Group (*N*)	Age Group	Dates 2025	*N* (%) Contacted	Cumulative *N* (%) of Total Responses
Initial invitation	Postal letter	All sampled individuals (16,005)	All	21 March	14,990 (93.7)	3437 (44.1)
First follow-up	Text via Heilsuvera	Non-responders (11,871)	All	3 April	10,806 (91)	6268 (80.5)
Second follow-up	Text message	Non-responders (2204)	18–60 years	6 May	2094 (95)	7314 (93.9)
	Telephone call	Non-responders (4439)	60–80 years	29 April–30 May	1222 (27.5)	
Third follow-up	Text message	a. Non-responders not yet contacted three times (3455)	18–80 years	12 June	3156 (91.3)	7674 (98.6)
	Telephone call	b. Non-responders (961)	81–85 years	27 June–15 July	808 (84.1)	
Data collection closed				6 November		7786 (100)

Note. Coverage varies across contact modes. Some postal invitations were undeliverable due to address inaccuracies, text messages via Heilsuvera were limited to individuals with a registered mobile number, and not all telephone contact attempts were successful due to unavailable numbers or unanswered calls.

**Table 4:** Comparison of demographic characteristics between responders and non-responders among cancer survivors and population controls.

	Cancer Survivors			Population Controls		
Variable	Responders *n* (%)	Non-Responders *n* (%)	*p*-Value	Responders *n* (%)	Non-Responders *n* (%)	*p*-Value
***N***	5489 (54.9)	4516 (45.1)	<0.001	2297 (40.6)	3366 (59.4)	<0.001
**Gender**			<0.001			0.824
Female	2970 (54.1)	2214 (49.0)		1181 (51.4)	1742 (51.8)	
Male	2518 (45.9)	2302 (51.0)		1116 (48.6)	1624 (48.2)	
Non-binary	1 (0.0)	0 (0.0)		0 (0.0)	0 (0.0)	
**Age group (years)**			<0.001			<0.001
19–39	207 (3.8)	202 (4.5)		60 (2.6)	196 (5.8)	
40–49	385 (7.0)	273 (6.0)		151 (6.6)	264 (7.8)	
50–59	794 (14.5)	518 (11.5)		341 (14.8)	472 (14.0)	
60–69	1648 (30.0)	971 (21.5)		722 (31.4)	814 (24.2)	
70–79	1865 (34.0)	1366 (30.2)		761 (33.1)	979 (29.1)	
80–99	589 (10.7)	1186 (26.3)		262 (11.4)	641 (19.0)	
**Region of residence**			0.051			0.014
Eastern Region	140 (2.6)	109 (2.4)		64 (2.8)	111 (3.3)	
Capital Region	3459 (63.0)	2912 (64.5)		1394 (60.7)	2027 (60.2)	
Northern Region	642 (11.7)	469 (10.4)		291 (12.7)	371 (11.0)	
Southern Region	547 (10.0)	423 (9.4)		226 (9.8)	329 (9.8)	
Southern Peninsula	321 (5.8)	308 (6.8)		141 (6.1)	258 (7.7)	
Moved abroad	3 (0.1)	6 (0.1)		1 (0.0)	16 (0.5)	
Westfjords	96 (1.7)	60 (1.3)		44 (1.9)	58 (1.7)	
Western Region	280 (5.1)	229 (5.1)		136 (5.9)	196 (5.8)	
**Cancer group**			<0.001			
Other	2443 (44.5)	2274 (50.4)				
Prostate	1061 (19.3)	873 (19.3)				
Breast	1395 (25.4)	831 (18.4)				
Colorectal	589 (10.7)	538 (11.9)				

Note. *p*-values are based on chi-square tests comparing responders and non-responders within each study group.

**Table 5:** Factors associated with study participation: Binary logistic regression analyses among cancer survivors and population controls.

	Cancer Survivors		Population Controls	
Variable	OR (95% CI)	*p*-Value	OR (95% CI)	*p*-Value
**Gender**				
Female	1.00 (Ref.)		1.00 (Ref.)	
Male	0.86 (0.78–0.96)	0.007	0.99 (0.88–1.10)	0.795
Non-binary	Not included		Not included	
**Age group (years)**				
19–39	0.81 (0.66–1.00)	0.049	0.39 (0.29–0.53)	<0.001
40–49	1.01 (0.85–1.21)	0.868	0.74 (0.59–0.92)	0.008
50–59	1.08 (0.95–1.24)	0.240	0.93 (0.78–1.10)	0.401
60–69	1.24 (1.11–1.38)	<0.001	1.15 (1.00–1.32)	0.047
70–79	1.00 (Ref.)		1.00 (Ref.)	
80–99	0.36 (0.32–0.40)	<0.001	0.52 (0.43–0.61)	<0.001
**Region of residence**				
Eastern Region	1.09 (0.84–1.42)	0.504	0.82 (0.59–1.12)	0.221
Capital Region	1.00 (Ref.)		1.00 (Ref.)	
Northern Region	1.15 (1.01–1.31)	0.038	1.14 (0.96–1.35)	0.131
Southern Region	1.08 (0.94–1.25)	0.269	0.96 (0.79–1.15)	0.628
Southern Peninsula	0.83 (0.70–0.98)	0.029	0.74 (0.59–0.91)	0.006
Moved abroad	0.36 (0.07–1.38)	0.151	0.08 (0.00–0.40)	0.015
Westfjords	1.32 (0.95–1.86)	0.104	1.04 (0.69–1.55)	0.849
Western Region	1.07 (0.89–1.29)	0.463	1.00 (0.79–1.26)	0.974
**Cancer group**				
Other	1.00 (Ref.)			
Prostate	1.25 (1.10–1.41)	<0.001		
Breast	1.45 (1.29–1.63)	<0.001		
Colorectal	1.12 (0.98–1.29)	0.093		

Note. OR = odds ratio; CI = confidence interval. Odds ratios are from binary logistic regression models predicting participation (responder vs. non-responder). Reference categories are indicated as “Ref.”. The non-binary gender category was excluded from regression analyses due to small numbers (*n* = 1).

**Table 6:** Participant-level survey completion (≥1 assigned instrument).

Completion Threshold	Participants (*N* = 7786)	%
100% completion	7330	94.1
≥80% completion	7626	98.0
≥50% completion	7668	98.5

**Table 7:** Instrument-level survey completion (all administered instruments).

Completion Threshold	Instruments (*N* = 20,927)	%
100% completion	15,032	71.9
≥80% completion	20,022	95.7
≥50% completion	20,294	97.0

## Data Availability

The data presented in this study are not publicly available due to legal and ethical restrictions related to the protection of personal data under the General Data Protection Regulation (GDPR) and national data protection legislation. Aggregated, non-identifiable data may be made available for research purposes upon reasonable request and subject to institutional approval and data minimization principles.

## Abbreviations

The following abbreviations are used in this manuscript:

CI	Confidence Interval
EORTC	European Organization for Research and Treatment of Cancer
HLS-EU-Q16	European Health Literacy Survey Questionnaire (16-item version)
HRQoL	Health-Related Quality of Life
ICR	Icelandic Cancer Registry
OR	Odds Ratio
PRO	Patient-Reported Outcomes
QLQ-BR42	Quality of Life Questionnaire Breast Cancer Module (42-item)
QLQ-BR-SURV40	Breast Cancer Survivorship Module (40-item)
QLQ-C30	Core Quality of Life Questionnaire (30-item)
QLQ-CR29	Colorectal Cancer Module (29-item)
QLQ-CR-SURV33	Colorectal Cancer Survivorship Module (33-item)
QLQ-PR25	Prostate Cancer Module (25-item)
QLQ-PR-SURV19	Prostate Cancer Survivorship Module (19-item)
QLQ-SURV100	Quality of Life Cancer Survivorship Questionnaire (100-item)
STROBE	Strengthening the Reporting of Observational Studies in Epidemiology
SURV-ICE	Survey of Cancer Survivorship in Iceland
